# Sustainable Synthesis of New Antioxidants from Hydroxytyrosol by Direct Biocatalytic Esterification in Ionic Liquids

**DOI:** 10.3390/molecules29215057

**Published:** 2024-10-26

**Authors:** Susana Nieto, Inmaculada Lozano, Francisco J. Ruiz, Jose F. Costa, Rocio Villa, Pedro Lozano

**Affiliations:** Departamento de Bioquímica y Biología Molecular B e Inmunología, Facultad de Química, Universidad de Murcia, E-30100 Murcia, Spain; inmaculada.lozanol@um.es (I.L.); franciscojavier.ruizm@um.es (F.J.R.); jf.costarubio@um.es (J.F.C.); rocio.villa@um.es (R.V.)

**Keywords:** lipases, bioactive molecules, tailor synthesis, neoteric solvents, nutraceuticals

## Abstract

Hydroxytyrosol (HT) is a nutraceutical compound, mainly found in the fruit, leaves and waste from the olive oil industry, known for exhibiting one of the highest antioxidant activities among molecules of natural origin. To harness this bioactivity in cosmetics, pharmaceuticals and the food industry, it is essential to modify the hydrophilicity of HT to enhance its compatibility with lipid-based mixtures. This chemical modification must be carried out with high selectivity to avoid compromising its radical scavenging activity. This work presents a highly efficient and selective approach to perform the biocatalytic esterification of free fatty acids (FFAs) of different alkyl chain lengths with HT in a reaction medium based on the SLIL [C_12_mim][NTf_2_]. By using a 1:2 (mol/mol) HT:FFA mixture of substrates, the HT-monoester derivative was obtained up to 77% yield after 2 h at 80 °C. The optimized molar ratio of substrates, combined with the ability to recover the SLIL for further reuse, significantly reduces waste accumulation compared to other reported strategies and results in a more sustainable approach as demonstrated by different green metrics. The antioxidant activity of HT-monoester products was fully maintained with respect to that presented by the natural HT, being stable for at least 3 months at 4 °C, as demonstrated by the DPPH and FRAP antioxidant analysis.

## 1. Introduction

Nature is a great supplier of chemical compounds with valuable antioxidant properties, being necessary to highlight the aromatic ones because of their ability to filter UV radiation and even counteract free radicals [1]. In particular, hydroxytyrosol (HT) is present in olive leaves and by-products resulting from olive processing (e.g., alperujo, etc.), being easily extracted from these renewable resources. As a bioactive compound, HT shows one of the highest antioxidant activities among other natural antioxidants (e.g., up to 10 times higher than catechols and 15 times higher than Coenzyme Q) [2]. This is related to the existence of two hydroxyl groups that are placed in an *ortho* position in the aromatic ring, improving the efficiency of donating electrons to free radicals due to the stabilization of the phenoxy group through hydrogen bonding [3,4,5]. As a result of this excellent antioxidant activity, HT is also included in the list of natural additives to prevent lipid rancidity in food products by either the direct addition to the lipid matrices or the incorporation into films for food preservation [2,6,7,8]. Moreover, the direct consumption of HT, as a key component of many nutraceutical preparations (e.g., cooking oils, beverages, dairy products, etc.), is related to anti-inflammatory or anti-diabetic effects, as well as the prevention of neural and cardiovascular degeneration [3,5]. In the same context, the cosmeceutical market is also interested in the use of bioactive ingredients for innovative formulations that are more efficient in preventing aging and wound healing [9].

The hydrophilic character of HT is a limiting issue for the preparation of cosmeceutical and nutraceutical products based on hydrophobic matrices, as well as favoring its adsorption and bioavailability [10]. Among other synthetic approaches, the biocatalytic esterification of fatty acids with HT appears as the most simple and clean approach to transform HT into a ‘phenolipid’ derivative [11].

Because of the mutual immiscibility of hydrophilic HT and free or esterified fatty acids as hydrophobic compounds, the use of volatile organic solvents (e.g., dimethyl carbonate, methyl tert-butyl ether and tert-butanol) as reaction media has been a common practice, despite their non-sustainable character [10,12].

As an alternative, ionic liquids (ILs) are exceptional non-aqueous reaction media for carrying out biocatalytic processes because of their unique array of physical–chemical properties (e.g., low vapor pressure, non-flammable nature, high ionic conductivity, good dissolution power towards many substrates, high thermal and chemical stabilities, etc.), as well as for preserving the activity and enhancing the stability of enzymes [13]. Furthermore, certain hydrophobic ILs with a sponge-like behavior, so-called sponge-like ionic liquids (SLILs), have allowed the design of efficient strategies for biocatalytic esterification that comprise a clean methodology for the separation of pure products [14]. This feature makes these SLILs highly convenient for the synthesis of food or cosmetic ingredients like flavors [15], monoacylglycerides of saturated or ω-3 fatty acids [13,16], panthenol esters [17] or monoesters of (hydroxy)cinnamic acids [18].

**Figure 1:**
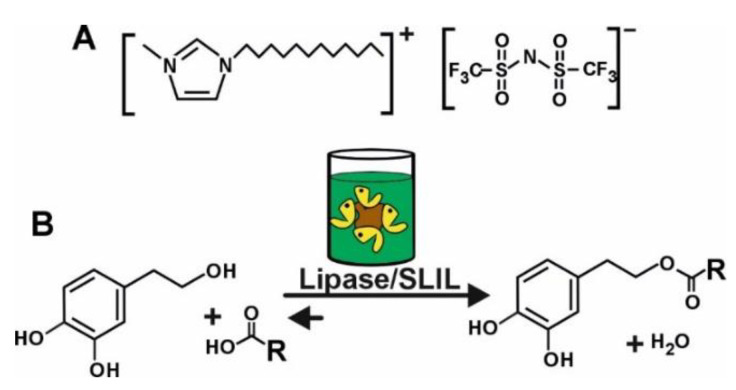
(**A**) Structure of the 1-dodecyl-3-methylimidazolium bistriflimide [C_12_mim][NTf_2_] SLIL; (**B**) Biocatalytic synthesis of hydroxytyrosyl monoesters by direct esterification of FFAs with HT in SLIL-based reaction media.

For the first time, this work shows an efficient and sustainable strategy for carrying out the biocatalytic synthesis of HT monoesters through the direct esterification of free fatty acids (FFAs) having alkyl-chain lengths ranging between C_6_ and C_18_, with HT in SLIL-based reaction medium (Figure 1). Direct esterification has been carefully selected as a cleaner and cost-effective synthetic strategy with respect to transesterification approaches because of the reduction in the accumulated by-products. The excellent synergy of SLILs and biocatalysts for the synthetic reaction, as well as the clean and straightforward product separation, is shown. The resulting HT monoesters showed the same antioxidant power as that of free HT. Compared to other conventional synthetic strategies previously reported, the higher sustainability of the biocatalytic process presented here is clearly demonstrated through different green metric parameters.

## 2. Results and Discussion

### 2.1. Suitability of IL-Lipase Combined Tools for the Esterification of FFAs with HT

The biocatalytic esterification of two substrates mutually immiscible, such as an aromatic alcohol, like HT, and an FFA as an acyl donor, may be considered the main handicap for a good performance, which is usually overcome by using chemical derivatives of substrates [19] or a great excess of inert solvents [20] to facilitate the reaction. By using ILs, many biocatalytic esterification reactions have been successfully carried out, where the easy recovery for reuse of this solvent was the main flag of greenness. As a representative example, the hexanoic acid (Hex) was selected as an acyl donor to carry out the biocatalytic esterification with HT by using a 1:4 HT:Hex molar ratio in the SLIL [C_12_mim][NTf_2_] as a green solvent. After the addition of the immobilized *C. antarctica* lipase B Novozym 435 (N435) (400 mg/mmol HT), the mixture was incubated at 80 ^°^C under magnetic stirring, where the presence of the MS 13X dehydrating agent allowed the reaction equilibrium to shift towards the synthetic side by withdrawing the released water by-product. Under these conditions, the hydroxytyrosyl hexanoate (HT-Hex) product was obtained at 87% yield after 3 h reaction time, as determined by HPLC (see Table 1, entry 4). The synthesis of the product was also confirmed by ATR-FTIR, revealing the formation of an ester bond through the identification of the C=O stretching by the shift of the vibration band of the carboxyl group from 1704 cm^−1^ to 1735 cm^−1^ and the detection of a new band at 1238 cm^−1^ corresponding to the C-O-C stretching (see Figure S1). In the same context, the HT-Hex product was identified and characterized by HPLC-MS, ^1^H-NMR and ^13^C-NMR analyses, as detailed in Supplementary Material (Figures S2–S7). The ^1^H-NMR and ^13^C-NMR spectra clearly showed that the primary OH group in the alkyl chain is the only one involved in the ester product, confirming the selectivity of the enzymatic esterification. 

These results show the effectiveness of the combination of biocatalysis and SLILs to achieve the efficient esterification of FFAs with HT. While the exquisite selectivity of the enzymes simplifies and improves the efficiency of this desired transformation, the selection of this SLIL as a non-aqueous green solvent with an excellent solubilization capacity permits us to significantly reduce its contribution to the mass transfer and improves the reaction rate compared to other organic solvents (see Section 2.5, Table 3, entries 2 and 3) [15,21]. Moreover, the potential for recovery and reuse of this SLIL is much more interesting from the economic and environmental points of view. Thus, it has been demonstrated that the synergy between the IL and the biocatalysts is fundamental for the efficient modification of natural bioactive compounds following the selectivity and economy criteria [13,14,15,16]. However, the excellence of this combo of IL-biocatalysts can be boosted through the optimization of the reaction conditions, attending to the amount of biocatalyst, the reaction temperature and the molar ratio of substrates. 

As can be seen in Table 1 (entries 1–4), the increase in the enzyme amount from 50 to 400 mg provides a concomitant increase in product yield up to 87% (see entry 4). However, it should be noted that the productivity of the reaction systems shows a bell-shaped profile as the amount of enzyme increases, the best results being obtained when using 100 mg N435/mmol HT (see entry 3), a value four times higher than that obtained for the highest enzyme content. Reaction temperature was also shown as an important parameter, being observed a clear improvement in product yield (from 47% to 83%, see entries 2, 5 and 6) when the temperature raised from 60 to 80 °C. This fact was directly related to the greater suitability of the reaction system for dissolving both substrates (HT and Hex) into the ionic net and enhancing their transfer rate to the active site of the enzyme whose activity is maintained by the protective effect of SLIL media [15]. The ability of hydrophobic SLILs to stabilize enzymes at high temperatures has been widely reported (e.g., up to 1370 days half-life time at 60 °C in the *N*-octadecyl-*N*’,*N*”,*N*”’-trimethylammonium bis (trifluoromethylsulfonyl)imide IL) [14,22]. This stabilization is attributed to the preservation of the essential water shell around the enzyme within the IL network, which acts as a stabilizing confined space for biocatalyst conformation. It should be noted that by decreasing the HT:Hex molar ratio to 1:2 mol/mol (see entry 7), both product yield (76%) and productivity (2.6 mmol HT-Hex/g N435 h) parameters remained practically similar to those obtained for a 1:4 HT:Hex molar ratio (see entry 2). Consequently, the 1:2 HT-Hex molar ratio was selected for further experiments because of the improvement in green metric parameters of the process (e.g., atom economy) [11,23].

### 2.2. Biocatalytic Synthesis of HT Monoesters with Different Acyl Chain Length

To analyze the suitability of the N435/[C_12_mim][NTf_2_] system for the lipophilization of HT, the biocatalytic synthesis of HT monoesters from fatty acids with different alkyl chain length was studied under the optimized reaction conditions.

**Figure 2:**
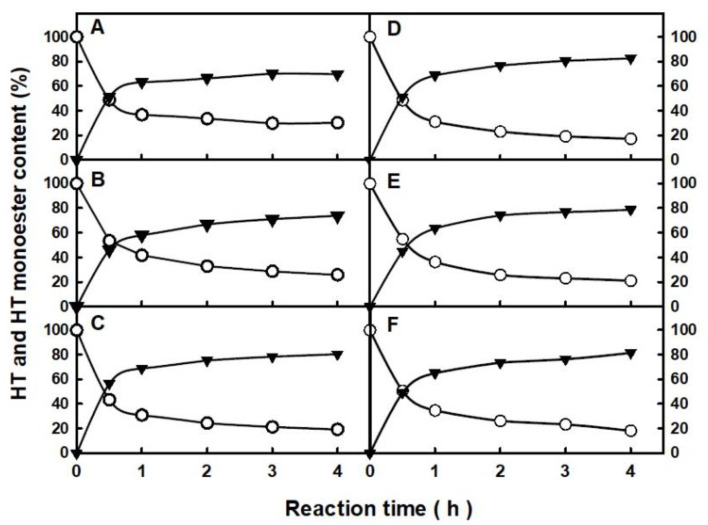
Time-course profiles of the enzymatic esterification of hexanoic acid, (**A**); octanoic acid, (**B**); decanoic acid, (**C**); lauric acid, (**D**); myristic acid; (**E**) and oleic acid, (**F**) with HT in [C_12_mim][NTf_2_] reaction medium. Free HT (○); HT-monoester product (▼).

Figure 2 shows the time-course profiles of the *N*-435-catalyzed direct esterification of hexanoic (C6), octanoic (C8), decanoic (C10), lauric (C12), myristic (C14) or oleic (C18:1) acid with HT using a 1:2 HT:FFA molar ratio at 80 °C. For all the assayed reaction systems, the immobilized enzyme was able to achieve a product yield higher than 50% within the first 30 min, whatever the size of the alkyl chain, reaching almost the maximum value in 2 h with a slight increase afterwards (i.e., up to 70–83% HT-monoester yield after 4 h reaction). The greater efficiency of the proposed approach can be observed after a comparative analysis with other strategies previously described, where similar yields were achieved after 16 h reaction by using a transesterification approach of synthesis in acetonitrile as reaction medium [19]. According to the profiles in Figure 2, a reaction time of 2 h was selected as the most appropriate for enzymatically producing HT monoesters, attending to the balance between high yield and productivity, as well as minimizing any possible undesired oxidations on HT derivatives induced by heat, that could occur after long reaction times at 80 °C.

**Figure 3:**
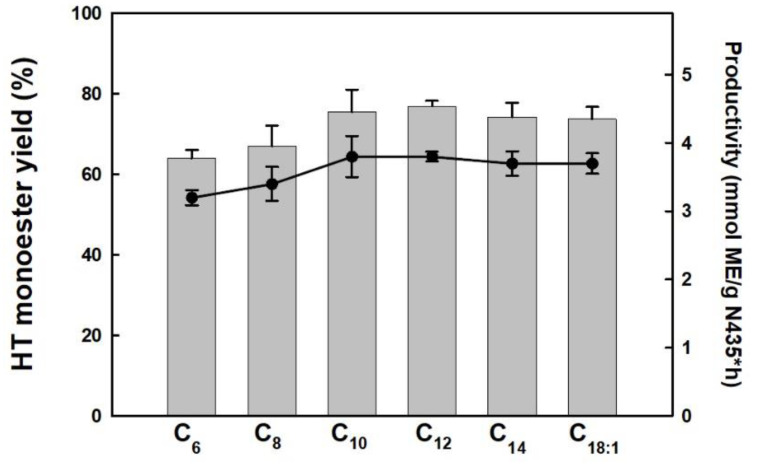
Yield (bars) and productivity (●) for the synthesis of HT-monoester derivatives by direct biocatalytic esterification of different FFAs (i.e., hexanoic acid, C_6_; octanoic acid, C_8_; decanoic acid, C_10_; lauric acid, C_12_; myristic acid, C_14_; oleic acid, C_18:1_) with HT. Reaction conditions: HT:FFA 1:2 (mol:mol), 100 mg N435/mmol HT, 2 h, 80 °C.

Figure 3 shows the evolution of the HT-monoester product yield and the productivity of the reaction system as a function of the alkyl chain length of the carboxylic acid used for the biocatalytic esterification of HT after 2 h of reaction. Both parameters show a similar behavior being increased with the alkyl chain length from hexanoic (C_6_) to lauric (C_12_) acids and then remained practically unchanged (e.g., approx. 80% yield and 3.8 mmol HT monoester/gN435·h) for myristic and oleic acids as the most hydrophobic cases. Although differences in the performance between the more hydrophobic FFAs were scarce, it can be noted that a slight improvement is obtained with lauric acid. A similar pattern was reported for the N435-catalyzed esterification of FFAs with different alkyl length with glycerol [13] and panthenol [17,23], where lauric acid also displayed the best performance for both, either in SLIL or solvent-free reaction media. For these hydrophobic SLIL-based reaction media, these results clearly demonstrated that the biocatalysts’ performance in the reaction system is positively influenced by the increase in the alkyl chain length of the acyl donor. This improvement is driven by the hydrophobic interactions between the alkyl chains of the FFA and the SLIL [C_12_mim][NTf_2_], fully aligning with the “like-dissolves-like” principle [14,22]. Thus, the best results obtained for lauric acid may be attributed to a favored mass-transfer rate of this fatty acid inside the IL net having the same alkyl chain length, as it was also reported for the case of the biocatalytic synthesis of aliphatic esters in IL/supercritical CO_2_ biphasic systems [24]. It is important to note that, despite the excess of acyl donor respect HT and the increased hydrophobicity of the reaction media with all tested FFAs, the degree of selectivity towards the synthesis of HT monoesters is not affected, not detecting diester products that could signify a decrease in the antioxidant activity. These results emphasized the excellent synergies between biocatalysts and SLILs to achieve the efficient and selective lipophilization of aromatic alcohols [11,14].

### 2.3. Scaling up of the Production of HT-Monohexanoate

**Figure 4:**
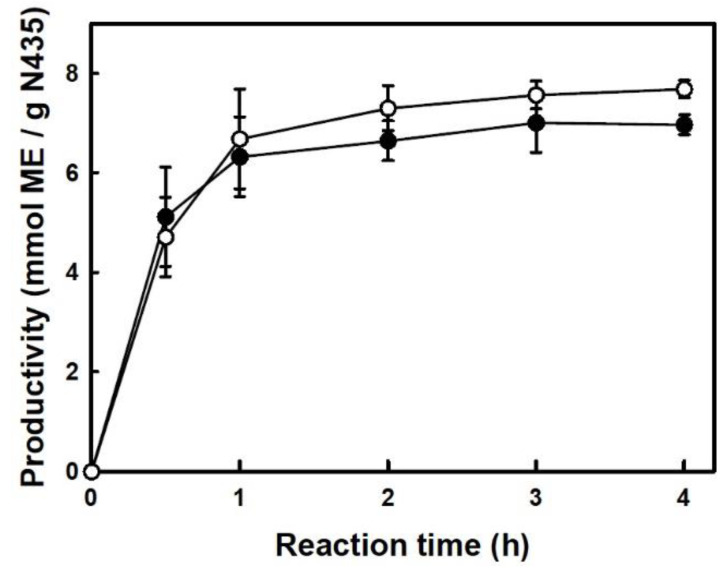
Time-course profiles of the accumulated productivity for the N435-catalyzed esterification of Hex with 0.5 mmol HT (●) or 5 mmol HT (○) (HT:Hex 1:2 mol:mol) using 100 mg N435/mmol HT in 70% (*w/w*) [C_12_mim][NTf_2_] at 80 °C.

To demonstrate the robustness of this procedure, a tempting assay to measure the suitability for scaling up was carried out by increasing 10-folds the reaction mass with respect to the optimized reaction in Table 1. Under these conditions, the incubation was performed in a reactor with an anchor mechanical stirring, coupled to a vacuum system to remove the water by-product released from the enzymatic reaction. Figure 4 shows the accumulated productivity (in terms of mmol of HT-monohexanoate per gram of N435) for the N435-catalyzed direct esterification of Hex with 0.5 mmol (low scale) or 5 mmol (high scale) HT by using a 1:2 HT:FFA molar ratio in 70% (*w/w*) [C_12_mim][NTf_2_] at 80 °C. The comparison of the time-course profiles reveals a similar biocatalytic performance in both scales of synthesis and even a slight improvement when the reaction mass is increased 10-fold, reaching a value close to 8 mmol HT-monohexanoate/g N435 at 3 h reaction time. This result was attributed to the better suitability of the mechanical anchor stirring for mixing the resulting viscous reaction medium with respect to the magnetic stirring used for low reaction size. By this approach, an adequate mass-transfer rate during the biocatalytic process occurred, as well as an efficient removal of the water by-product produced along the reaction by the vacuum system coupled to the reactor. These results highlight the relevance of the setup as an additional element to the N435/SLIL system to achieve the best performance. The intensification of the biocatalytic synthesis provided by the suitable setup at high reaction volumes was also observed, although to a greater extent, for the synthesis of panthenyl monolaurate [23] and xylityl monolaurate [25] by direct esterification in solvent-free media.

**Figure 5:**
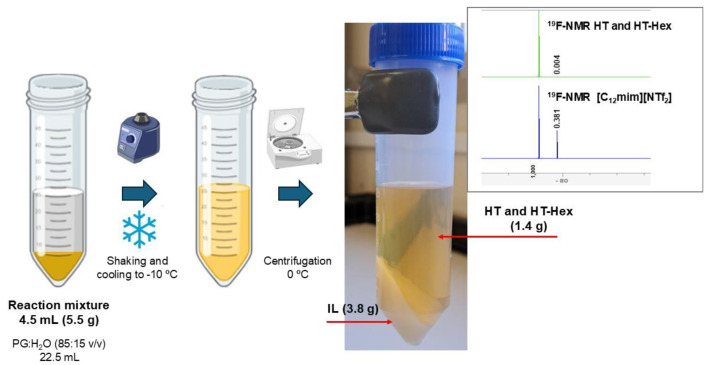
Scheme of the downstream process to extract the HT-Hex product. The mass balance along the reaction is indicated, as well as the phase behavior of the reaction mixture after adding five volumes of propylene glycol:H_2_O (85:15 *v/v*), then cooling at −10 °C and centrifugation (10 min, 0 °C, 14,000 rpm). The supernatant containing the extracted products was analyzed by ^19^F-NMR using the IL [C_12_mim][NTf_2_] as a control reference. The clear SLIL can be reused for another operational cycle. Created with BioRender.com.

To build green chemical processes, it is necessary to develop integrated approaches for selective (bio)transformation and separation, capable of providing the products directly and the recovery for reuse of all the elements of the reaction system (e.g., biocatalysts, solvents, etc.). The SLILs (e.g., [C_12_mim][NTf_2_], etc.) are temperature-switchable ionic liquid/solid phases that behave as sponge-like systems that permit us to develop straightforward and clean approaches for product separation after the biocatalytic step by a simple protocol of cooling and centrifugation. By this approach, the SLIL precipitates as a solid salt at the bottom, while the products remain in the upper liquid phase, as pure products when they are liquids or dissolved in another green molecular cosolvent (e.g., water, etc.) previously added [14,15,17,22]. For this system, the full release of HT-monohexanoate and unreacted HT from the IL matrix could not be achieved by simple cooling and centrifugation of the reaction mixture, as occurred for volatile flavors [15]. For the proper extraction of these compounds, the addition of propylene glycol:water (PG:H_2_O, 85:15 *v/v*) green solution was necessary, providing a liquid–liquid biphasic system. This mixture was then fully shaken, cooled and centrifuged to precipitate the solid SLIL (bottom phase), while the released HT and HT-monohexanoate were accumulated in the propylene glycol:water phase (upper phase, see Figure 5). Briefly, five volumes of PG: H_2_O (85:15 *v/v*) were added to 4.5 mL (4.5 g) of the reaction mixture from the biocatalytic step for HT-monohexanoate synthesis in [C_12_mim][NTf_2_]. From the initial mass of 4.5 g, a total of 1.35 g made of HT-Hex (94%) and unreacted HT (6%) were recovered in the liquid phase. A ^19^F-NMR analysis (insert in Figure 5 and Figure S8) showed the presence of traces of SLIL (up to 1%) that can be fully eliminated by other classical procedures (i.e., ionic exchange column). The precipitated SLIL accounted for 4.2 g and its white color (identical to the pure SLIL) points to almost the total release of the reaction species. Only a content of 10% HT-Hex was still retained in the ionic net, having the possibility to carry out another purification step or to directly reuse this SLIL in another operational cycle. 

It should be noted that propylene glycol (PG) meets the specifications of the Food Chemicals Codex (Report Number: 27 NTIS Accession Number: PB265504, 1973) in agreement with the Select Committee on GRAS Substances (SCOGS) [26]. Because of the safety of PG, the extracted mixture containing the HT monoester could be used without additional steps of purification, contrary to other strategies where the use of volatile organic solvents involves tedious workups that reduce the greenness of the overall process and increase waste.

### 2.4. Antioxidant Activity of the HT-Monoesters

The industrial interest in preparing lipophilized HT derivatives to be used as nutraceuticals in hydrophobic-based formulations is fully dependent on the maintenance of the antioxidant power with respect to the free HT. The antioxidant activity of HT and its derivatives after the esterification with different alkyl-chain length FFAs was determined by their capacity to reduce the free radical 2,2-diphenyl-1-picrylhydrazyl, which manifests through a color turn from deep purple to pale yellow that can be quantified by Vis-UV spectroscopy at 517 nm. [11,27,28] To carry out this assay, SLIL-free samples were first obtained from the reaction media by liquid–liquid extraction, and their respective concentrations were determined by HPLC using a calibration pattern. Free HT and vitamin C (ascorbic acid) were used as control references of the antioxidant activity, and the concentration of all samples was adjusted to 80 nmol for a proper comparison (see Materials and Methods section). The analysis was performed on a freshly extracted reaction mixture and, after 3 months of storage at 4 °C, was run in duplicate to obtain the mean and standard deviation. Additionally, the capacity of the different HT monoesters for reducing the Fe^3+^-specie was determined by means of the FRAP method (see Table 2). 

**Figure 6:**
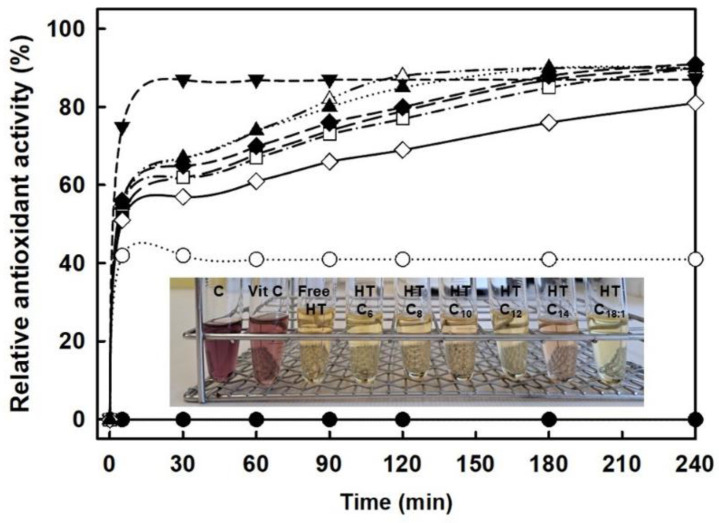
Time-course profiles of the relative antioxidant activity of free HT (▼) and HT monoesters based on different alkyl-chain lengths (C_6_, △; C_8_, ■; C_10_, □; C_12_, ◆; C_14_, ♢; C_18:1_, ▲; overall content in HT species: 80 nmol), determined spectrophotometrically at 517 nm during 4 h with DPPH. Vitamin C (80 nmol, ○) was used as an antioxidant standard reference. A sample of DPPH without any antioxidant (●) was used as a control to establish the zero value. Insert picture: final color displayed by each sample after the DPPH test.

According to Table 2, the results obtained by the DPPH assay showed that the relative antioxidant activities of HT and HT monoesters ranged from 89 ± 3.7 to 95 ± 0.5%, being clearly higher than that resulted from the same concentration of vitamin C (49.3 ± 13%). This could be attributed to the double reductive moieties onto the HT structure with respect to the sole reductive group of vitamin C [29]. Furthermore, it should be noted how all the HT-monoesters derivatives maintain the same antioxidant activity, regardless of the length of the alkyl chain of the FFA, since the selective esterification did not affect the aromatic hydroxyl groups, activity as demonstrated by NMR analysis (see Figure S7). However, attending to the time-course profile of the DPPH reduction (Figure 6), a certain slowdown in the reaction rate was detected for HT esters with respect to free HT and vitamin C. This fact had already been reported for the lipophilization of HT [20,30], as well as for other aromatic acids (e.g., caffeic acid, coumaric, etc. [11]), being attributed to a lower ionization of the aromatic hydroxyl groups after the esterification. This slowdown could be responsible for the decay in the antioxidant activity measured at short reaction times (<1 h) in the DPPH method [28,31]. Additionally, the DPPH assay of the different HT monoesters stored at 4 °C for three months showed a similar antioxidant capacity, highlighting the stability of the HT monoesters.

Alternatively, an FRAP assay was also used to determine the antioxidant activity of the HT monoesters (see Materials and Method section). The obtained results confirmed a high antioxidant power of these HT monoesters, obtained values between 1.4 and 4.9 µM TE per each nanomole of the sample analyzed, which was also maintained after 3 months at 4 °C. These results push again on the excellent suitability of these compounds as antioxidants. It should be noted that despite the study of the antioxidant stability of HT monoesters is limited due to the short time elapsed, the obtained results are promising for the use of these products as stable antioxidant additives in food preservation conditions.

Thus, the esterification of FFAs with HT not only improves the miscibility with lipophilic formulations in cosmetics or foods, favoring skin penetration and intestinal absorption but also the longer reaction time of HT-monoester derivatives could be considered an advantage to extend the antioxidant activity over time. Thus, the combination of these HT monoesters with other antioxidants with a faster rate (i.e., vitamin C or free HT) could be interesting as a protective system for providing a fast and more prolonged answer against free radicals.

### 2.5. Green Metric Assessment of the Biocatalytic Synthesis of HT-Monoesters

To assess the sustainability of the biocatalytic strategy here presented, the synthesis of HT-monohexanoate was selected as a representative example of HT monoesters. Using different recognized green metrics, i.e., atom economy (AE), yield (ε), stoichiometric factor (SF), mass recovery parameter (MRP), reaction mass efficiency (RME), process mass intensity (PMI), E-factor and total carbon release (TCR) parameters and the Ecoscale tool, a “gate to gate” analysis has been made comprising the synthesis and workup. The AE, 1/SF and ε parameters provide information about the reactivity of substrates and atoms incorporated into the desired products. It should be noted that the MRP concerns the recyclability of the reaction species (or their contribution to waste), whereas RME is considered a global indicator of sustainability, comprising all the above parameters. The values of all these parameters range between zero to one, corresponding with the highest value to the best sustainability. Alternatively, the E-factor parameter may be used as waste quantification criteria, being expected to have the lowest value for sustainable processes. This E-factor can be extrapolated from the RME or the PMI, being the last parameter used to identify the origin of waste (see Table S1) and the sensitive steps of further optimization to promote waste reduction. Also, the PMI permits us to calculate the emissions of CO_2_ through the total carbon release (TCR) parameter, where a different factor is applied to organic (waste accumulated in the synthesis step) and aqueous (wastewater in the downstream steps) waste to determine the CO_2_ production, considering a scenario of waste incineration [32]. The EcoScale tool provides approximate information about the LCA by considering aspects of the toxicity and hazardousness of the reagents or the energy invested in the whole process of synthesis and purification (see Material and Methods and Supplementary Material sections for further details) [11,23].

For a comparative green analysis, other reported strategies for HT-monoester biocatalytic synthesis were selected as a result of the sufficient information they provided (see Table 3) [10,19,20]. Among other conditions, the most relevant differences between these approaches concern the use of volatile organic solvents as reaction medium (entries 2–4), as well as aliphatic esters (entries 1 and 2) as activated acyl donors for a transesterification approach, which provides easy solubilization into the solvent that resulted in homogeneous reaction media. In all cases, the reaction was catalyzed by N435, providing excellent yields (75–98%) but different performances. Thus, a high HT-monoester yield (93%, entry 2) was obtained at the shortest reaction time (1.25 h), leading to the highest value of productivity (5.4 mmol ME/g N435 h). These results are slightly higher than those here reported by a direct esterification approach (see entry 4, 3.1 mmol HT monoester/g N435∙h). It can be noted that the use of a very low amount of biocatalyst (33 mg/mmol HT, see entry 3) involves a lengthening in the reaction time up to 48 h for achieving a 75% HT-monoester yield, being the productivity greatly reduced (0.5 mmol ME/g N435 h). In this regard, it seems more appropriate to increase the amount of enzyme to improve esterification reaction rates and productivity.

**Figure 7:**
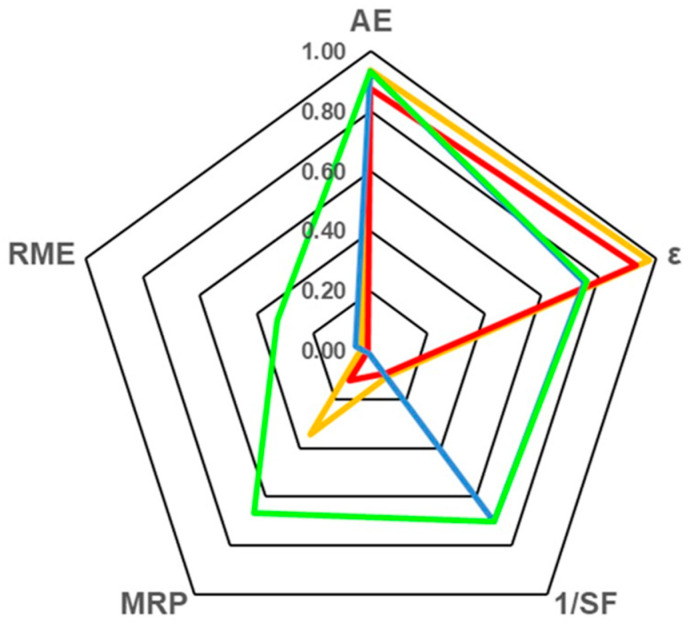
Radial pentagon of the green metric analyses for approaches included in Table 3. Orange, entry 1 [19]; red, entry 2 [20]; blue, entry 3 [10]; green, entry 4 [This work].

However, yield and productivity parameters only focus on reaction efficiency, and other critical aspects related to reaction conditions and preparation that provide information on resource use (substrates, energy, solvents, etc.) and waste generation must also be taken into account [11,23]. Therefore, a complementary sustainability analysis becomes necessary to identify the most efficient approach. In this regard, the ε was complemented with other green parameters like AE, 1/SF, MRP and RME, which are usually represented as the vertices of a pentagon (Figure 7). As can be seen, the greenness of the process is shown when a balanced pentagon with a maximum radius of one is obtained, which is the highest value for each metric [33]. The analysis of these metrics has been strictly referred to as the synthesis step (or upstream) to identify the most efficient reaction conditions.

The AE parameter provides information about the suitability of the strategy selected, revealing the contribution of by-products to the overall synthesis. Thus, the application of an esterification strategy (entries 3 and 4, Table 3; blue and green lines, Figure 7) shows the highest values of AE as a result of the release of water as a unique by-product. Although the transesterification strategy provides the release of alcohol as a by-product, the high AE value resulted in entries 1 and 2 is due to the lower molecular mass of ethanol and acetaldehyde by-products, respectively, with respect to the large alkyl chain of the obtained HT-monoester products. As the AE parameter does not consider the substrate’s mass balance in the reaction system, the green analysis must be complemented with the stoichiometric factor parameter (1/SF), which quantifies the excess of substrates with respect to the reaction stoichiometry. Then, an important decay in the 1/SF parameter was obtained for entries 1 and 2 (values of 0.1) due to the great presence of fatty acid esters used as acyl donors. On the contrary, the use of a 1:2 HT:Hex molar ratio (see blue and green lines, Figure 7) raises the 1/SF parameter to an acceptable value of 0.7. When using an excess of one of the substrates, the contribution to waste is great, being strongly penalized by other green metric parameters. For example, attending to the MRP formula in Table S1, this excess of substrates is quantified twice, in the 1/SF parameter and in the waste (W) term, having a profound impact on the reduction in the value of the MRP parameter. Also, the use of organic solvents as non-recoverable input or the low reaction yield greatly impacts a poor value of MRP. Conversely, the suitability of SLILs to be recovered and reused [19,20,21,28], together with the fair molar ratio of substrates used in the strategy here reported, leads to the best MRP value (0.66, entry 4), being 66-folds higher than the MRP value of entry 3 (0.01) despite using the same reaction conditions. The RME parameter collects the results of all the above green metrics to provide an overall landscape of the reaction sustainability and thus, may be considered as the most important parameter in the radial pentagon. As can be seen, whilst the reported strategies (see lines blue, orange and red) adopt a triangle or a square shape because their RME value is almost null (0.01–0.03), the direct esterification of Hex with HT in SLILs (green line) results in an RME value of 0.33, being the only one that fits a balanced pentagon and shows the higher values for almost all the assayed metric parameters.

**Figure 8:**
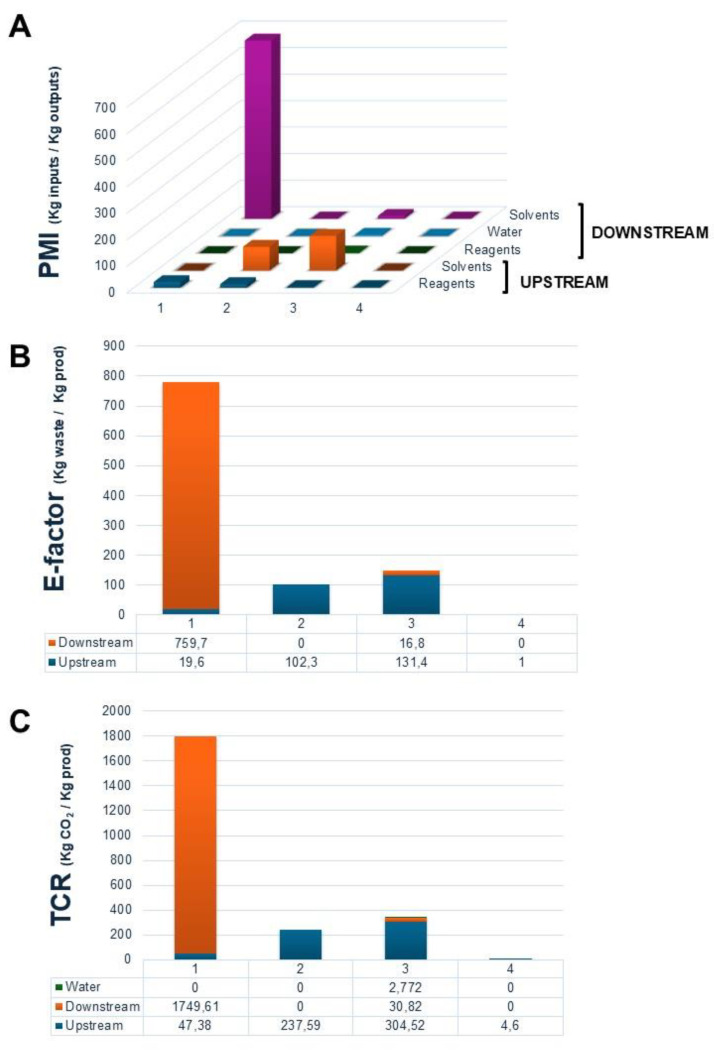
Analysis of waste and CO_2_ emissions in the strategies in Table 3. (**A**) PMI: Representation of the contribution of substrates and reagents, solvents and water to the PMI value in the upstream and downstream processes; (**B**) E-factor: Relation of mass of waste accumulated with respect to the mass of the HT-monoester product in the upstream and downstream processes; (**C**) TCR: Mass of CO_2_ emissions as a result of the incineration of organic and aqueous waste in the upstream and downstream processes. The analyses for entry 2 are only referred to the upstream.

Alternatively, the determination of the PMI parameter is useful to detect those steps that can be improved for reducing waste. The PMI calculator allows us to determine waste accumulation through the different steps of upstream and downstream. Because of the insufficient information on solvent mass used in the workup in entry 2, the complete analysis of the PMI parameter was only carried out for entries 1, 3 and 4 (see Table 3). Figure 8A provides the distribution of the source of waste for each of the selected strategies resulting from the PMI calculator. Although entry 1 is a solvent-free synthetic approach, the high mass of solvents used in the workup tarnishes its sustainability (up to 670 kg input/kg output). It should be noted how the use of a fully recoverable solvent, like SLIL, joined to the high biocatalytic efficiency achieved for entry 4, leads to the best PMI results.

From the PMI parameter, the E-Factor refers to the overall generated waste, while the TCR parameter points to the waste contribution to CO_2_ emissions (see Table S1 and Figure 8B,C) [34]. The results obtained for E-factor and TCR follow the same pattern as that observed for the PMI case, pointing again to the negative character of non-recoverable solvents to the environmental burden.

To obtain an overview of the LCA for the synthesis of HT monoesters according to the selected strategies, the analysis was complemented with the EcoScale. Although this tool does not allow for a fine discrimination between reaction conditions, it is very useful to evaluate certain parameters not contemplated in the previous metrics, such as the energy invested, the price and toxicity of the reagents or the stages and equipment necessary for the synthesis and workup. Thus, starting from an initial value of 100% sustainability, penalties are assigned as a function of those criteria that have been collected in detail in Table S2 (it should be remembered that the workup of entry 2 is incomplete). For entries 1–3, most of the penalties come from the cost and safety of substrates and reagents used. For example, the higher price of the activated acyl donor (i.e., ethyl palmitate and vinyl decanoate) is heavily penalized compared to FFAs. The lower yield obtained in entries 3 and 4 is also penalized, though this tool only considers the yield of the limiting substrate and does not account for the excess substrate used in entries 1 and 2. This is a clear example of the limitations of this tool, together with the rough discrimination between the reaction conditions in the section of technical setup. However, these weaknesses can be counterbalanced by combining this tool with other green metric parameters.

As a result, all the penalties assigned reduce the scores for entries 1–3 from 100% (corresponding to an ideal sustainable reaction) to almost 50%. Meanwhile, the one here reported obtains a value of 77% due to the lower range of reagents and the simplification and safety of the overall process. This punctuation, according to the authors of this tool, corresponds to excellent operating conditions [35].

In all the green parameters analyzed in this work, the contribution to waste of the SLIL used in this work has been omitted due to its recovery. However, the potential environmental impact of IL versus other volatile organic solvents can be discussed. So far, there are a limited number of LCA studies performed on ILs, which present certain gaps due to the lack of relevant information [36]. Regarding the case of [C_12_mim][NTf_2_] SLIL, its low vapor pressure prevents evaporation, while its high hydrophobicity also prevents mixing with aqueous media. This means greater control of its release compared to organic solvents and allows it to be recovered and reused almost entirely over several operational cycles before degradation.

## 3. Materials and Methods

### 3.1. Materials 

Commercial HT (2,4-dihydroxyphenylethanol, Naturolive HT15SF with 15% purity was a kind gift from Deretil Nature, S.A (Almeria, Spain). As a pure standard, a commercial HT (>98%) from TCI was also used. Sigma provided different free fatty acids: hexanoic acid (C6, 99%), octanoic acid (C_8_, >98%), decanoic acid (C_10_, ≥98%), lauric acid (C_12_,98%), mirystic acid (C_14_, 98%) and oleic acid (C_18:1_, 90%), as the dehydrating agent molecular sieves MS13X, acetophenone (99%), deuterated dimethyl sulfoxide (DMSO-δ_6_) and the free radical 2,2-DiPhenyl-1-PicrylHydrazyl (DPPH), while ascorbic acid (≥99%) was supplied by Probus, S.A. (Barcelona, Spain). IoLiTec (Ionic Liquids Technologies, Heilbronn, Germany) was the source of the IL 1-dodecyl-3-methylimidazolium bis(trifluoromethyl-sulfonyl)imide ([C_12_mim][NTf_2_], 99% purity). The commercial immobilized *Candida antarctica* lipase B, named Novozym^®^ 435 (N435), was a gift of Novozymes/Novonesis (Madrid, Spain).

### 3.2. Biocatalytic Synthesis of Hydroxytyrosol Esters 

The commercial product Naturolive HT15SF is a complex extract from *Olea europaea* containing 15–17% HT, 45–65% fruit extract (fats, sugars, dietary fiber and proteins) and 35–55% starch. Before the use, HT was extracted with methanol (10 g extract/60 mL MeOH) for 12 h, and afterwards, methanol was evaporated in a rotary evaporator at 60 °C and 110 rpm. The concentration of recovered HT was measured by HPLC with respect to a calibration pattern of pure HT (0.02–1 mM) from TCI (99% purity) using acetophenone (1 mM) as the internal standard, obtaining a concentration of 2.7 M. The biocatalytic esterification process of HT was carried out by using different FFAs as acyl donors (i.e., hexanoic acid, C_6_; octanoic acid, C_8_; decanoic acid, C_10_; lauric acid, C_12_; myristic acid, C_14_; oleic acid, C_18:1_). To perform the process, 1:4 or 1:2 molar ratios of HT and FFAs were dissolved in 0.5 mL of the SLIL-[C_12_mim][NTf_2_] containing MS 13X (100 mg/mmol HT). After mixing at 60–80 °C, 250 rpm, N435 (50–400 mg N435/mmol HT) was added, and the reaction was incubated in the same conditions for 8 h. Along the reaction, 10 μL aliquots were withdrawn at different intervals to obtain the time-course profiles through HPLC analysis. The higher scale reaction was performed by increasing 10-folds the overall mass in a Carousel Plus 6 Reaction Station system with a TornadoTM Overhead Stirring System (Radleys, Saffron Walden, United Kingdom) coupled to a vacuum system (Vacstar IKA, Barcelona, Spain).

The assays performed at benchmark or at higher scale were conducted in triplicate to obtain the mean and standard deviation. 

At the end of the biocatalytic reaction, the immobilized enzyme derivative was separated from the medium by centrifugation (i.e., 14,000 rpm, 15 min, RT). A liquid–liquid extraction procedure was developed to separate the HT monoesters and unreacted HT from the SLIL as follows. Approximately 200 µL of reaction medium were suspended in propylene glycol: H_2_O (85: 15 *v/v*, 1 mL) and stirred for 5 min at room temperature. The resulting heterogeneous mixture was cooled to −10 °C for 15 min and centrifuged at 0 °C (i.e., 14,000 rpm, 10 min), resulting in two separated phases: an upper liquid phase containing unreacted HT and HT-monoester products and a solid-white bottom phase corresponding to the SLIL. Residual SLIL on the liquid phase was determined by ^19^F-NMR, as follows. The sample (40 µL) and trifluoroacetic acid (TFA, 40 µL, internal standard) were dissolved in DMSO-δ_6_ (420 µL) and then analyzed in a Brucker AC 200E spectrometer 400 MHz (Massachusetts, United States, U.S.A.), quantifying the residual IL with respect to a standard of the [C_12_mim][NTf_2_] SLIL prepared in the same conditions.

### 3.3. HPLC Analysis

The separation and identification of substrates and products was performed by HPLC using an HPLC LC-20 system (Shimadzu, Columbia, MD, USA) coupled to a photodiode detector (SPD-M20A, Shimadzu), with an RP-18 column (LiChrospher, Merck, Darmstadt, Germany, 250 nm × 5 μm). The solvents acetonitrile (ACN, A) and orthophosphoric acid 0.1% *v/v* (B) were used according to the following elution gradient: 0–2 min, 25% A; 2–16 min, 25–90% A; 16–17 min, 90%; 17–18 min, 90–25% A, 18–25 min, 25% A. For the reaction with octadecenoic acid as an acyl donor, the gradient varied as follows: 0–2 min, 25% A; 2–16 min, 25%–90% A; 16–25 min, 90% A; 25–26 min, 90%–25% A; 26–32 min, 25% A. HT and the ester products were identified at their λ_max_ (280 nm) with the following retention times: HT (3.0 min), HT-C6 (13.6 min), HT-C8 (15.8 min), HT-C10 (17.8 min), HT-C12 (19.7 min), HT-C14 (21.4 min) andHT-C18:1 (23.4 min). The yield of the esterification was determined as a function of the peak area balance of HT and the ester product. 

### 3.4. FTIR Spectra

The vibration bands of the functional groups in HT, FFAs and the ester products were identified by infrared spectroscopy (FT-IR-4700 JASCO Analytical Instruments, Easton, PA, USA) with a range of measurement from 3500 to 400 cm^−1^, at a 0.4 cm^−1^ resolution.

### 3.5. HPLC-MS and NMR Analyses

The reaction model of HT esterification with hexanoic acid was selected to identify the ester product by HPLC-MS and NMR spectra. HPLC-MS analyses were performed with an HPLC-DAD Agilent 1200 equipped with an RP-C18 column (250 mm × 5 μm) and an electrospray detector ESI-TOF Agilent 6220 (Agilent, California U.S.A.) following the same elution gradient as in Section 2.3 but replacing orthophosphoric acid by acetic acid. Signals were obtained by scanning in the range of 100–1000 *m*/*z* operating in negative ion mode. The ion spectra were compared with a NIST library for the identification of the reaction species.

Equally, ^1^H-NMR and ^13^C-NMR spectra were obtained for HT, Hex, the SLIL-[C_12_mim][NTf_2_], and the reaction mixture with a Bruker Avance 400 MHz spectrometer. For the analyses, 50 μL were diluted with DMSO-δ_6_ up to a final volume of 400 μL.

Hydroxytyrosol: ^1^H-NMR δ(ppm)—3.48 (dt, 2H, H_a_); 4.53 (t, 1H, H_a_-OH); 2.52 (t, 2H, H_b_); 6.42 (dd, 1H, H_d_); 6.60 (d, 1H, H_e_); 8.57/8.67 (s, 1H, H_f_-OH or H_g_-OH, indistinguishable); 6.57 (d, 1H, H_h_). ^13^C-NMR δ(ppm): 62.6 (C_A_); 38.5 (C_B_); 130.1 (C_C_); 119.4 (C_D_); 116.3 (C_E_); 143.3 (C_F_); 144.9 (C_G_); 115.4 (C_H_). 

Hexanoic Acid—^1^H-NMR δ(ppm): 11.95 (s, 1H, H_i_-OH); 2.18 (t, 2H, H_j_); 1.48 (q, 2H, H_k_); 1.18–1.33 (m, 4H, H_l_ and H_m_, indistinguishable); 0.85 (t, 3H, H_n_). ^13^C-NMR δ(ppm): 174.5 (C_I_); 33.6 (C_J_); 24.2 (C_K_); 30.8 (C_L_); 21.9 (C_M_); 13.8 (C_N_). Hydroxytyrosyl Hexanoate—^1^H-NMR δ(ppm):4.11 (t, 2H, H_a_); 2.67 (t, 2H, H_b_); 6.43 (dd, 1H, H_d_); 6.59, (d, 1H, H_e_); 6.56 (d, 1H, H_h_); 2.23 (t, 2H, H_j_); 1.47 (q, 2H, H_k_); 1.18-1.33 (m, 4H, H_l_ and H_m_, indistinguishable); 0.85 (t, 3H, H_n_). ^13^C-NMR δ(ppm): 64.6 (C_A_); 33.8 (C_B_); 128.5 (C_C_); 119.4 (C_D_); 116.5 (C_E_); 143.6 (C_F_); 145.1 (C_G_); 115.7 (C_H_); 172.9 (C_I_); 33.5 (C_J_); 24.1 (C_K_); 30.7 (C_L_); 21.8 (C_M_); 13.8 (C_N_). 

1-Dodecyl-3-methylimidazolium bis(trifluoromethylsulfonyl)imide—^1^H-NMR δ(ppm): 4.14 (t, 2 H, H_a′_); 1.77 (q, 2 H, H_b′_); from 1.32 to 1.15 (m, 18 H, from H_c′_ to H_k′,_ indistinguishable); 0.85 (t, 2H, H_l′_); 9.09 (dd, 1H, H_m′_); 7.75 (dd, 1H, H_n′_); 7.76 (dd, 1H, H_o′_); 3.84 (s, 3H, H_p′_). ^13^C-NMR δ(ppm): 48.8 (C_A′_); from 29.3 to 28.4 (from C_B′_ to C_I′_, indistinguishable); 25.5 (C_C′_); 31.3 (C_J′_); 22.1 (C_K′_); 13.8 (C_L′_); 136.5 (C_M′_); 123.6 and 122.1 (C_N′_ and C_O′_, indistinguishable).

### 3.6. Antioxidant Activity of HT Monoesters by Radical Scavenging Test

The antioxidant activity of HT monoesters was determined by using the DPPH method [10,32,33]. First, samples of reaction media (200 μL) containing HT monoesters based on different FFA acyl donors (see Figure 3) were suspended in ethyl acetate (1 mL), and the mixture was strongly shaken for 10 min at RT to extract all HT compounds. Then, the ethyl acetate phase was collected, and the solvent was removed under reduced pressure. The remaining solid fraction was then dissolved in MeOH (0.5 mL) and the concentration of HT species was determined by HPLC, as described in Section 3.2. To determine the stability of HT-esters, fresh samples (recently extracted) or extracts that had been stored at 4 °C for 3 months were used. For all cases, concentrations ranged from 0.3 to 0.37 M HT species, where HT-monoester derivative accounted for 72–83%, maintaining the same proportion with free HT as in the reaction media. 

To perform the DPPH assay, all extracted fractions were diluted with methanol to achieve a 0.8 mM HT-monoester final concentration to determine the antioxidant activity. Samples of HT methanolic fraction (100 μL, 80 nmol) were added to 3 mL of 0.15 mM DPPH in MeOH, shaken at RT, and then the absorbance at 517 nm was recorded until it reached the steady state. The relative antioxidant activity, in terms of the capacity to neutralize the free DPPH radical, was calculated according to the formula:

(1) $$Relative\;Antioxidant\;Activity\left(\%\right)=\frac{A_{C}-A_{S}}{A_{S}}\times 100$$

where, A_C_ corresponds to the absorbance of the DPPH control solution (without antioxidant), and A_S_ corresponds to the absorbance of DPPH in the presence of the antioxidant sample.

Additionally, the FRAP method was used to determine the capacity of the samples to reduce the ferric ion of the complex 2,4,6-tripyridyl-s-triazine complex [Fe^3+^-(TPTZ)_2_]^3+^ to obtain a ferrous complex [Fe^2+-(^TPTZ)_2_]^2+^ with an intense blue color that is registered at 593 nm [37,38]. A calibration pattern with Trolox (50–500 µM) was used as a reference. A volume of 100 µL of each sample (50 µM) was used to determine this activity, being referred to as µM Trolox equivalents/nmol of the HT sample. 

The DPPH and FRAP studies were conducted in duplicate to obtain the mean and standard deviation.

### 3.7. Analysis of Sustainability

To assess the sustainability of the reaction, the efficiency and waste generation were determined by means of six green metric parameters as previously reported [29]: atom economy (AE), yield (ε), stoichiometric factor (SF), material recovery parameter (MRP), reaction mass efficiency (RME), process mass intensity (PMI), E-factor and TCR (see Section 3 in Supplementary Material, for more information). The PMI calculator was used to determine the PMI (https://www.acs.org/content/dam/acsorg/greenchemistry/industriainnovation/roundtable/convergent-pmi-tool.xlsx). The EcoScale tool was also used (http://ecoscale.cheminfo.org/calculator). 

## 4. Conclusions 

The current framework of sustainability in industrial processes demands a shift towards the use of renewable raw materials and the better use of resources to decrease the environmental burden. The increased interest in natural antioxidants for the cosmetic and food markets opens the necessity to develop highly selective, green and clean synthetic approaches for preparing new products with improved bioactivities. This means a turn of conventional approaches by introducing new tools that afford more sustainable and cost-effective processes. This work demonstrates how the combination of biocatalysis with the SLIL [C_12_mim][NTf_2_], as a neoteric green solvent, is a highly suitable approach for the selective preparation of lipophilic HT monoesters, preserving their bioactive properties. The synergy between the remarkable solvent capacity of this SLIL and the biocatalytic efficiency permits us to achieve high productivity (up to 3.8 mmol HT-monoester/g N435·h) in the synthesis of HT monoesters bearing different alkyl chain lengths, with the lowest waste accumulation compared to other strategies previously reported. Moreover, the interesting properties of SLILs have been key for the development of a clean, innocuous and simple extraction of the products, allowing for the direct application of the mixture because of the biological interest of non-reacted substrates and the GRAS solvent used. Thus, because of the antioxidant activity of HT monoesters and their tailored hydrophobicity, a more efficient use as a nutraceutical or antioxidant additive in the food sector is expected. Moreover, the combination of these HT monoesters with other bioactive molecules may lead to synergistic effects, improving the benefits of cosmetics or pharmaceutics formulations. For example, HT and panthenol (pro-vitamin B5) are bioactive molecules that share the fact that they have been esterified to reduce their hydrophilicity, but both also contribute to wound healing through the decrease in inflammation and the promotion of fibroblast proliferation, respectively. Thus, the combination of both bioactive ingredients could mean an improvement in the rate of wound healing.

However, more studies are necessary before implementing this technology in industry. So far, the synthesis of 1.4g of the product has been considered, but it is necessary to increase the scale of synthesis and assess the recyclability of the system SLIL/biocatalysts. Also, a long-term analysis of the stability of the antioxidant activity of the synthesized HT monoesters under different temperature conditions or after the incorporation into different matrices should be convenient to determine their industrial suitability compared to other known antioxidants. 

Once again, the synergic combination of biocatalysts and SLIL opens an interesting path to upgrade the greenness of chemical transformations for a more sustainable future.

## Figures and Tables

**Table 1:** Influence of reaction parameters on the biocatalytic synthesis of hydroxytyrosyl hexanoate by esterification of Hex with HT (0.25 mmol) in 70% (*w/w*) [C_12_mim][NTf_2_] reaction medium. Yield (ε) and productivity (mmol HT-Hex/g N435 h) have been calculated at 3 h reaction time.

Entry	HT:Hex (mol: mol)	N435/HT (mg/mmol)	T (^°^C)	ε (%)	Productivity (mmol HT-Hex/g N435 h)
1	1:4	50	80	33	2.2
2	1:4	100	80	83	2.8
3	1:4	200	80	81	1.4
4	1:4	400	80	87	0.7
5	1:4	100	70	71	2.4
6	1:4	100	60	47	1.6
7 ^a^	1:2	100	80	78	2.6

^a^ 0.5 mmol HT.

**Table 2:** Antioxidant activity of HT and HT monoesters by DPPH and FRAP. Fresh samples or samples stored for 3 months at 4 °C were analyzed. The results of the DPPH assays are expressed as a percentage of relative antioxidant activity with respect to the blank methanol. The ferric-reducing activity determined by FRAP is expressed as µM Trolox equivalent/nmol of sample). N.D.: Not determined.

Entry	DPPH Relative Antiox. Activ. (%)		FRAP (µM TE/nmol Sample)	
	Fresh	Stored 4 °C	Fresh	Stored 4 °C
Vit C	49.3 ± 12.7	N.D.	N.D.	N.D.
HT	92.9 ± 0.0	89.8 ± 3.7	2.0 ± 0.1	N.D.
HT-C6	93.1 ± 3.3	92.7 ± 3.3	1.5 ± 0.0	2.2 ± 0.2
HT-C8	95.6 ± 0.1	92.7 ± 3.6	1.6 ± 0.2	2.3 ± 0.1
HT-C10	95.5 ± 0.8	92.2 ± 3.4	2.4 ± 0.0	1.9 ± 0.0
HT-C12	95.9 ± 0.3	92.7 ± 3.1	2.2 ± 0.1	2.0 ± 0.0
HT-C14	88.7 ± 12.0	72.9 ± 3.1	1.9 ± 0.1	1.4 ± 0.0
HT-C18:1	95.4 ± 0.5	92.8 ± 3.5	2.9 ± 0.3	4.9 ± 0.0

**Table 3:** Reaction conditions and analysis of sustainability of different biocatalytic strategies for hydroxytyrosol lipophilization.

	Entry	1, [19]	2, [20]	3, [10]	4, [This work] ^a^
**Reaction conditions**	Solvent (mL/mmol HT)	None	MTBE ^b^ (35)	MTBE (33)	[C_12_mim][NTf_2_] (5)
	Acyl donor	Ethyl palmitate	Vinyl decanoate	Hexanoic acid	Hexanoic acid
	HT (mmol)	0.4	0.72	6	5
	HT:DA (mol:mol)	1:30	1:20	1:2	1:2
	mg N435/mmol HT	100	139	33	100
	Temperature (°C)	37	40	40	80
	Time (h)	4	1.25	48	3
	ε (%)	98	93	75	76 ± 1.4
	Productivity (mmol HT ester/g Enz h)	2.5	5.4	0.5	3.1 ± 0.2

^a^ From this work, the biocatalytic esterification reaction of Hex (10 mmol) with HT (5 mmol) was selected for green metric analysis; ^b^ MTBE, methyl *tert*-butyl ether.

## Data Availability

Data are contained within the article and Supplementary Materials.

## Supplementary Materials

The following supporting information can be downloaded at https://www.mdpi.com/article/10.3390/molecules29215057/s1, Figure S1. FTIR spectra of HT, Hex, HT-Hex ester and the LI-[C12mim][NTf2]; Figure S2. HPLC-MS analysis in negative ion mode of the esterification of HT with hexanoic acid; Figure S3. ^1^H-NMR (A) and ^13^C-NMR (B) spectra of commercial hydroxytyrosol (TCI); Figure S4. ^1^H-NMR (A) and ^13^C-NMR (B) spectra of hydroxytyrosol used in this work, kindly gifted by Deretil Nature S.A.; Figure S5. ^1^H-NMR (A) and ^13^C-NMR (B) spectra of hexanoic acid; Figure S6. ^1^H-NMR (A) and ^13^C-NMR (B) spectra of IL [C12mim][NTf2]; Figure S7. ^1^H-NMR (A) and ^13^C-NMR (B) spectra of the reaction medium of HT-Hex synthesis; Figure S8. ^19^F-NMR spectra of [C12mim][NTf2] and the extracted sample of HT-Hex; Table S1. Green Metric Parameters, equations, and definitions [39]; Table S2. List of penalties assigned in each category of the EcoScale.

## Abbreviations

AE, atom economy; E-factor, environmental factor; FFA, free fatty acid; Hex, hexanoic acid; HT, hydroxytyrosol; MRP, material recovery parameter; PG, propylene glycol; PMI, process Mmss Intensity; RME, reaction mass efficiency; SLIL, sponge-like ionic liquid; SF, stoichiometric factor; TCR, total carbon release; TE: Trolox equivalents.
