# Supplementary material for: Sustainable Synthesis of New Antioxidants from Hydroxytyrosol by Direct Biocatalytic Esterification in Ionic Liquids
Source: Molecules. 2024 Oct 26;29(21):5057. doi: 10.3390/molecules29215057 (PMC11547527; doi:10.3390/molecules29215057)
Supplement: Supplementary file 1 [file molecules-29-05057-s001.zip › molecules-3239106-supplementary.pdf]

Supplementary Material

Sustainable synthesis of new antioxidants from hydroxytyrosol by direct biocatalytic esterification in ionic liquids

Susana Nieto*, Inmaculada Lozano, Francisco J. Ruiz, Jose F. Costa, Rocío Villa, Pedro Lozano*.

Departamento de Bioquímica y Biología Molecular B e Inmunología. Facultad de Química, Universidad de Murcia, E-30100 Murcia, Spain

* Corresponding author. To whom all the correspondence should be addressed.

1. Confirmation of the synthesis of hydroxytyrosyl hexanoate.

1.1. FTIR analyses

The esterification of HT with hexanoic acid was selected a reaction model to test the suitability of the biocatalytic approach developed in ILs. FTIR analyses were conducted to identify the vibration bands of the functional groups in HT, FFAs and the ester products using a FT/IR-4700 (JASCO Analytical Instruments, Easton, PA, EE.UU.) with a range of measurement 3,500 - 400 cm^{-1} and 0.4 cm^{-1} resolution.

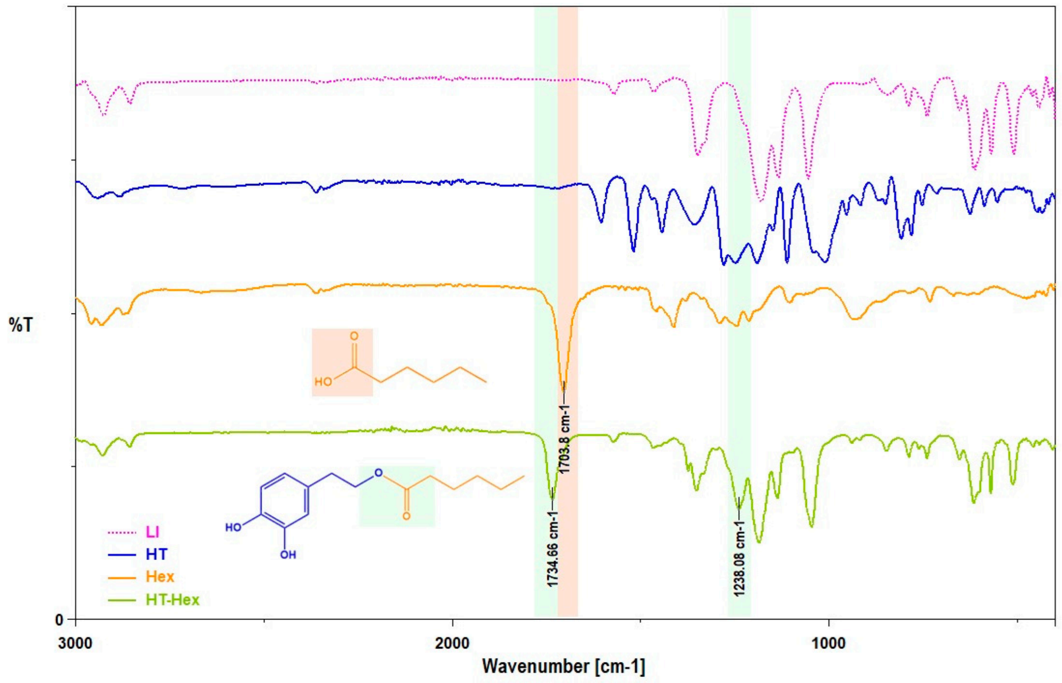

Figure S1. FTIR spectra of HT, Hex, HT-Hex ester and the LI-[C_{12}mim][NTf_2].

The shift of the vibration band of the carbonyl group from 1704 cm^{-1} to 1735 cm^{-1} is due to the stretching provoked by the ester bond. The new C-O-C bond also leads to the detection of a new band at 1238 cm^{-1}.

1.2. HPLC-MS analysis

HPLC-MS analyses were performed with a HPLC-DAD Agilent 1200 equipped with a RP-C18 column (250 mm × 5 μm) and an electrospray detector ESI-TOF Agilent 6220 (Agilent, USA). Signals were obtained by scanning in the range 100-1000 m/z operating in negative ion mode. The ion spectra were compared with a NIST library for the identification of the reaction species (Figure S2).

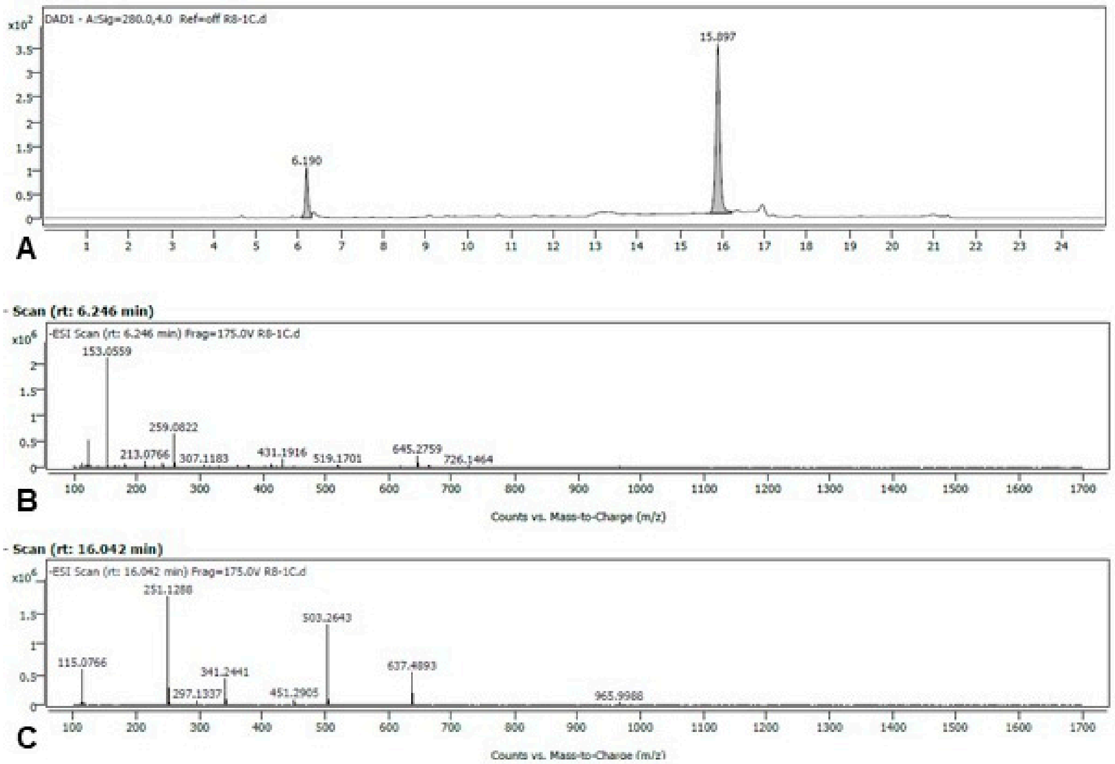

Compound Summary

Cpd	Formula	RT	Mass	ID Source	Score
1	C8 H10 O3	5.698	154.0630	FBF	99.90
2	C14 H20 O4	15.932	252.1362	FBF	99.23

D

Figure S2. HPLC-MS analysis in negative ion mode of the esterification of HT with hexanoic acid. A. Peaks of HT (6.2 min) and HT-Hex (15.9 min) detected at 280 nm. B. ESI chromatogram of the peak at 6.2 min retention time containing ions with m/z=153, corresponding to HT. C. SI chromatogram of the peak at 15.9 min retention time. Ions

with m/z=251 are detected, corresponding to HT-Hex. D. Table of compounds identification.

The absorption chromatogram at 280 nm reveals two peaks (Figure S2.A), the first one corresponding to non-reacted HT (m/z=154, Figure S2.B) and a more important peak corresponding to HT-Hex (m/z= 252, Figure S2.C).

1.3. NMR analyses

Additionally, ^{1}H-NMR and ^{13}C-NMR assays provide relevant information about the synthesis and structure of HT-Hex. Samples of 50 µL of HT, Hex, [C_{12}mim][NTf_2] and the reaction media containing HT-Hex, were diluted with DMSO-δ6 up to 400 µL final volume and analyzed in a Bruker Avance, 400 MHz spectrometer.

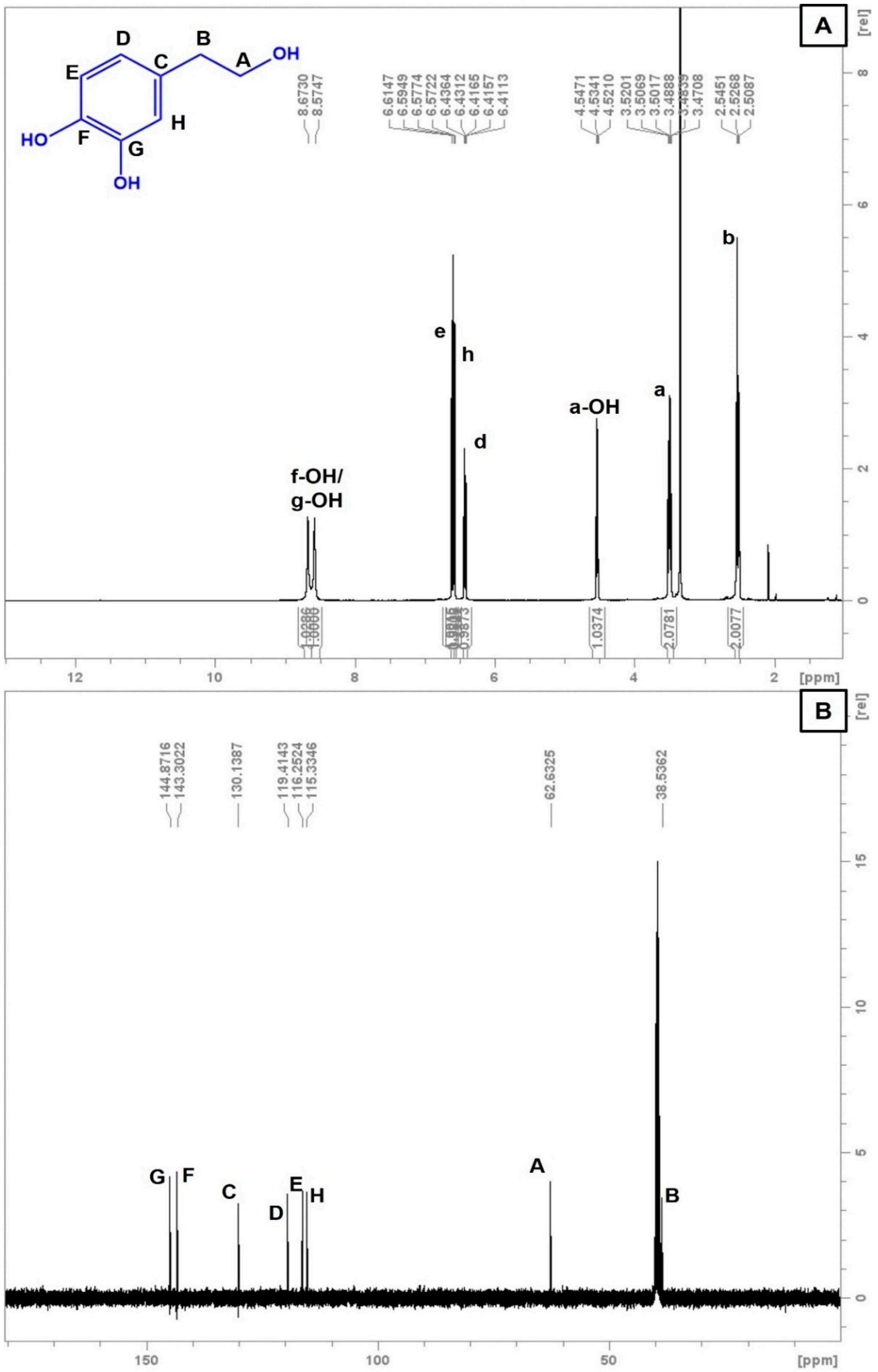

Figure S3. ^{1}H-NMR (A) and ^{13}C-NMR (B) spectra of commercial hydroxytyrosol (TCI).

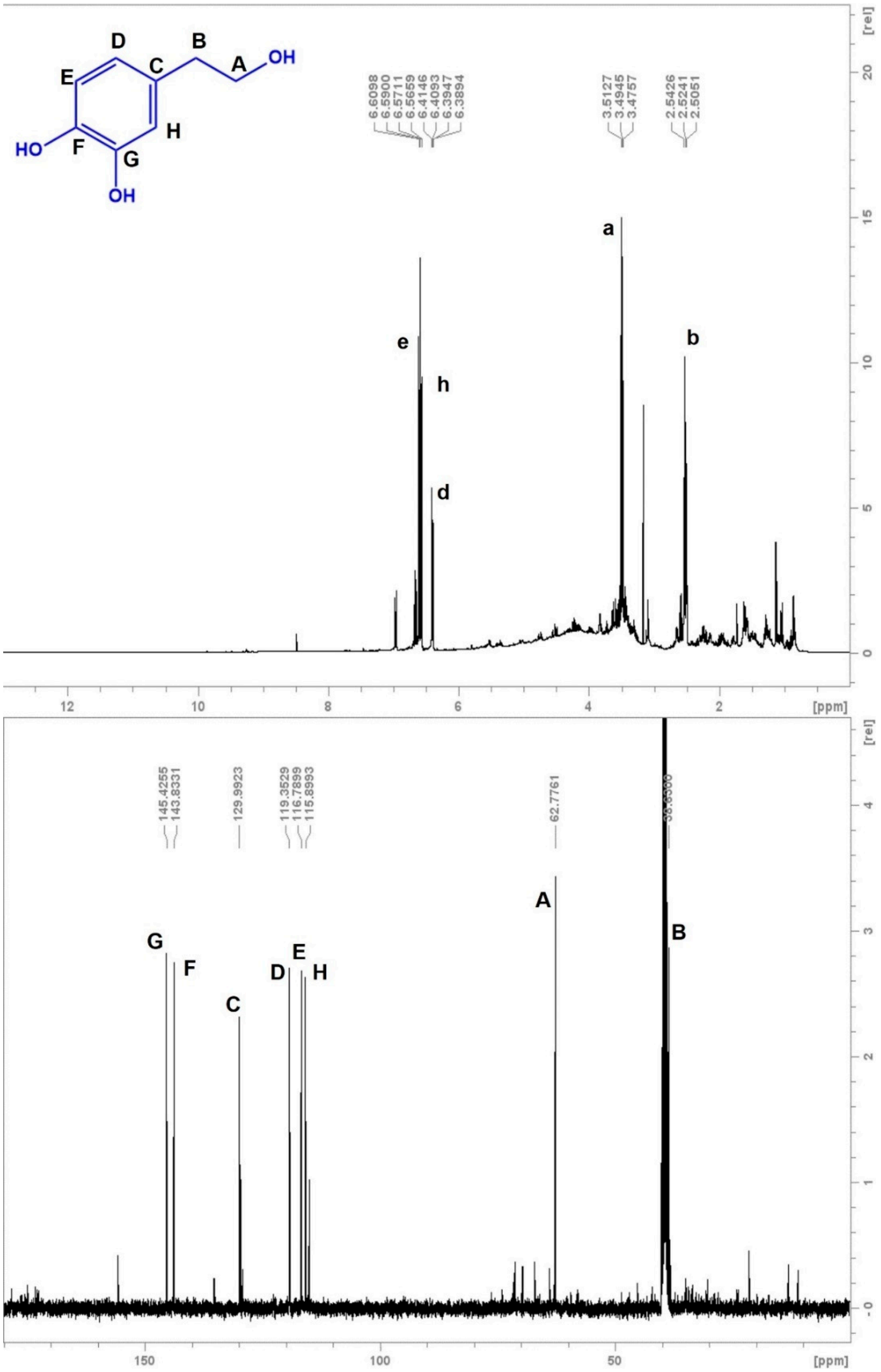

Figure S4. ^{1}H-NMR (A) and ^{13}C-NMR (B) spectra of hydroxytyrosol used in this work, kindly gifted by Deretil Nature S.A.

Hydroxytyrosol: ^{1}H-NMR δ(ppm): 3.48 (dt, 2H, H_a); 4.53 (t, 1H, H_a-OH); 2.52 (t, 2H, H_b); 6.42 (dd, 1H, H_d); 6.60 (d, 1H, H_e); 8.57/8.67 (s, 1H, H_f-OH or H_g-OH, indistinguishable); 6.57 (d, 1H, H_h). ^{13}C-NMR δ(ppm): 62.6 (C_A); 38.5 (C_B); 130.1 (C_C); 119.4 (C_D); 116.3 (C_E); 143.3 (C_F); 144.9 (C_G); 115.4 (C_H).

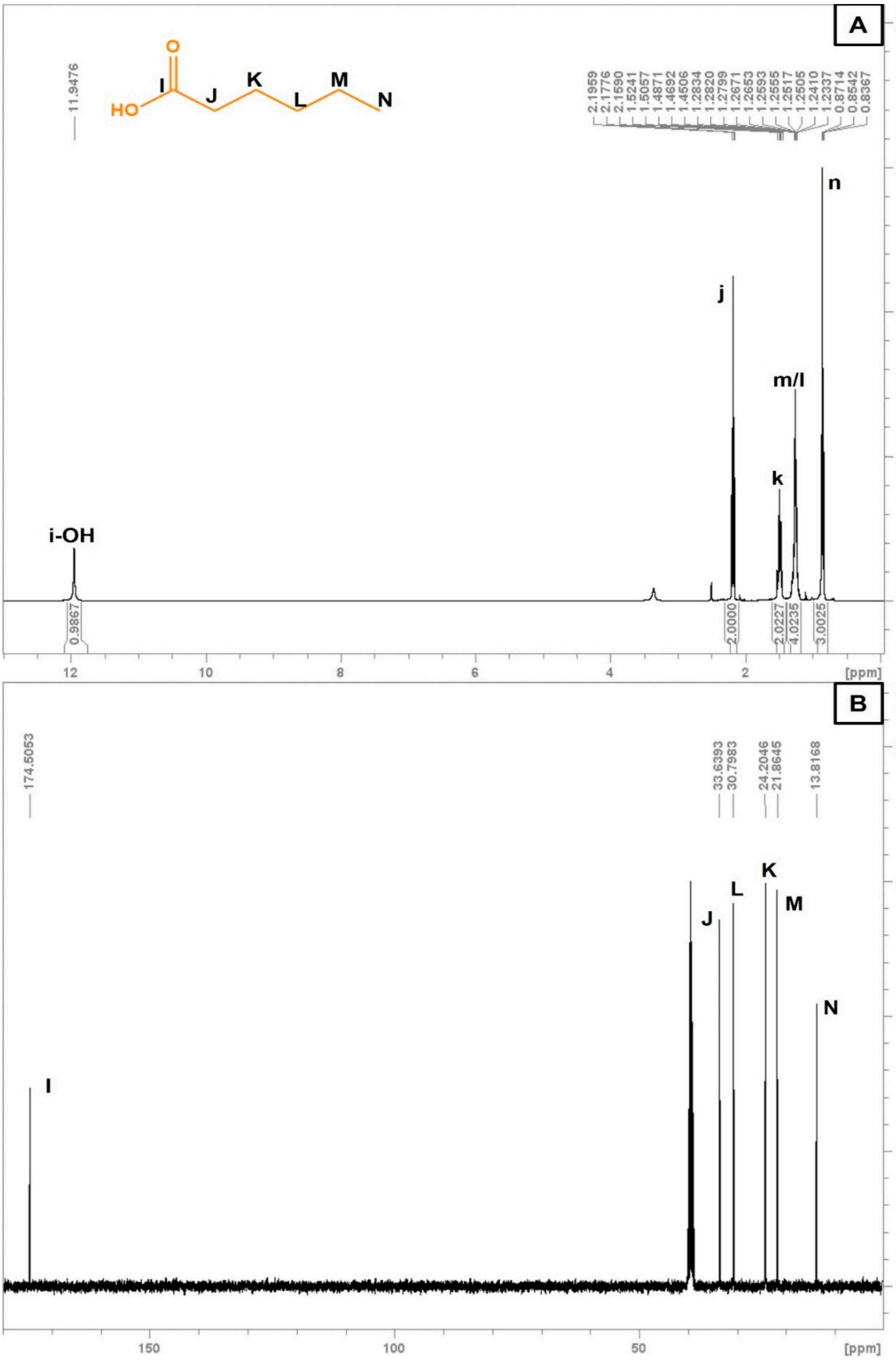

Figure S5. ^{1}H-NMR (A) and ^{13}C-NMR (B) spectra of hexanoic acid.

Hexanoic acid: ^{1}H-NMR δ(ppm): 11.95 (s, 1H, H_i-OH); 2.18 (t, 2H, H_j); 1.48 (q, 2H, H_k); 1.18-1.33 (m, 4H, H_l and H_m, indistinguishable); 0.85 (t, 3H, H_n). ^{13}C-NMR δ(ppm): 174.5 (C_I); 33.6 (C_J); 24.2 (C_K); 30.8 (C_L); 21.9 (C_M); 13.8 (C_N).

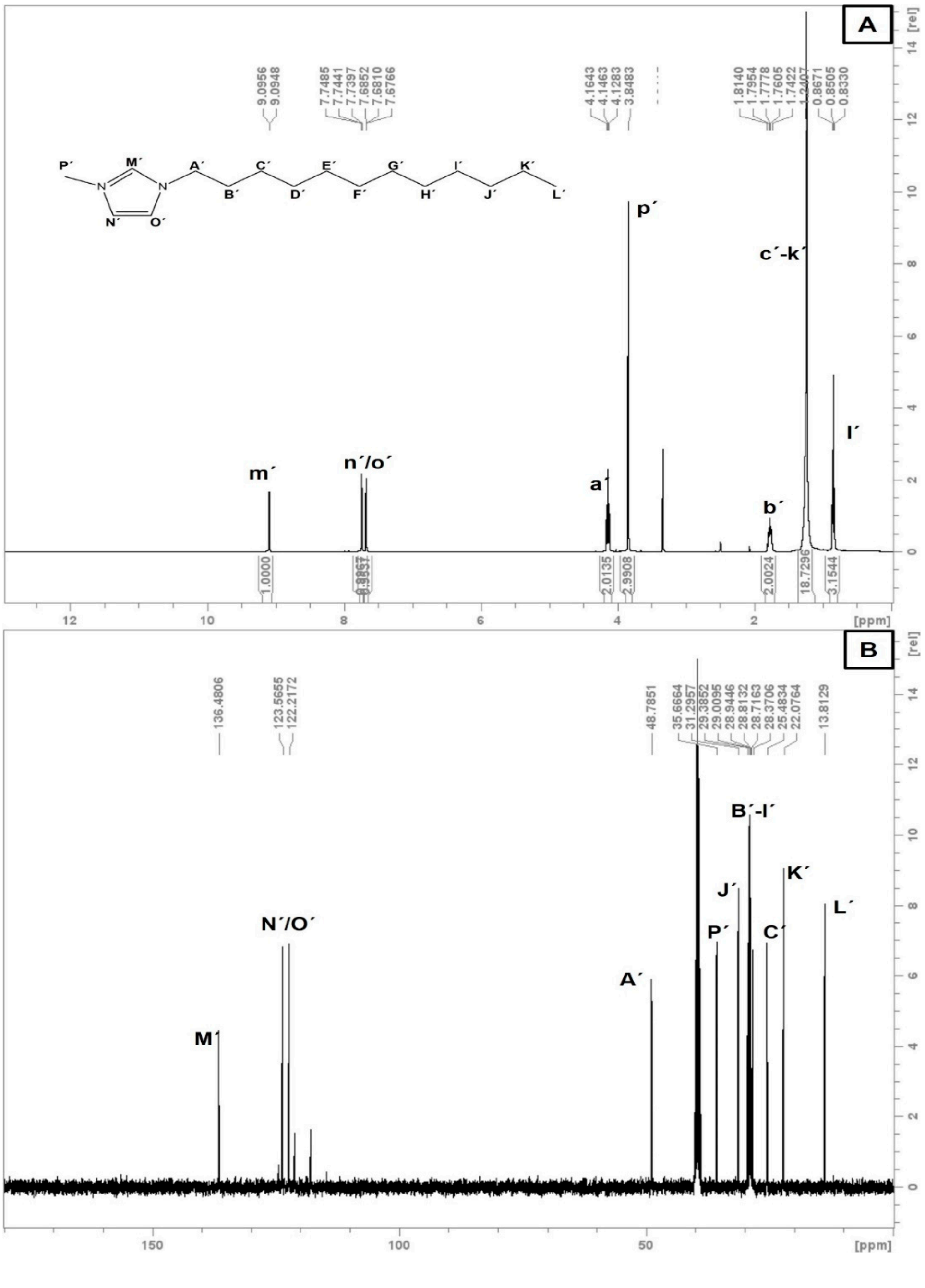

Figure S6. ^{1}H-NMR (A) and ^{13}C-NMR (B) spectra of IL $[C_{12}mim][NTf_2]$.

1-Dodecyl-3-methylimidazolium bis(trifluoromethylsulfonyl)imide: ^{1}H-NMR δ(ppm): 4.14 (t, 2 H, $H_{a'}$); 1.77 (q, 2 H, $H_{b'}$); from 1.32 to 1.15 (m, 18 H, from $H_{c'}$ to $H_{k'}$, indistinguishable); 0.85 (t, 2H, $H_{l'}$); 9.09 (dd, 1H, $H_{m'}$); 7.75 (dd, 1H, $H_{n'}$); 7.76 (dd, 1H, $H_{o'}$); 3.84 (s, 3H, $H_{p'}$). ^{13}C-NMR δ(ppm): 48.8 ($C_{A'}$); from 29.3 to 28.4 (from $C_{B'}$ to $C_{I'}$, indistinguishable); 25.5 ($C_{C'}$); 31.3 ($C_{J'}$); 22.1 ($C_{K'}$); 13.8 ($C_{L'}$); 136.5 ($C_{M'}$); 123.6 and 122.1 ($C_{N'}$ and $C_{O'}$, indistinguishable).

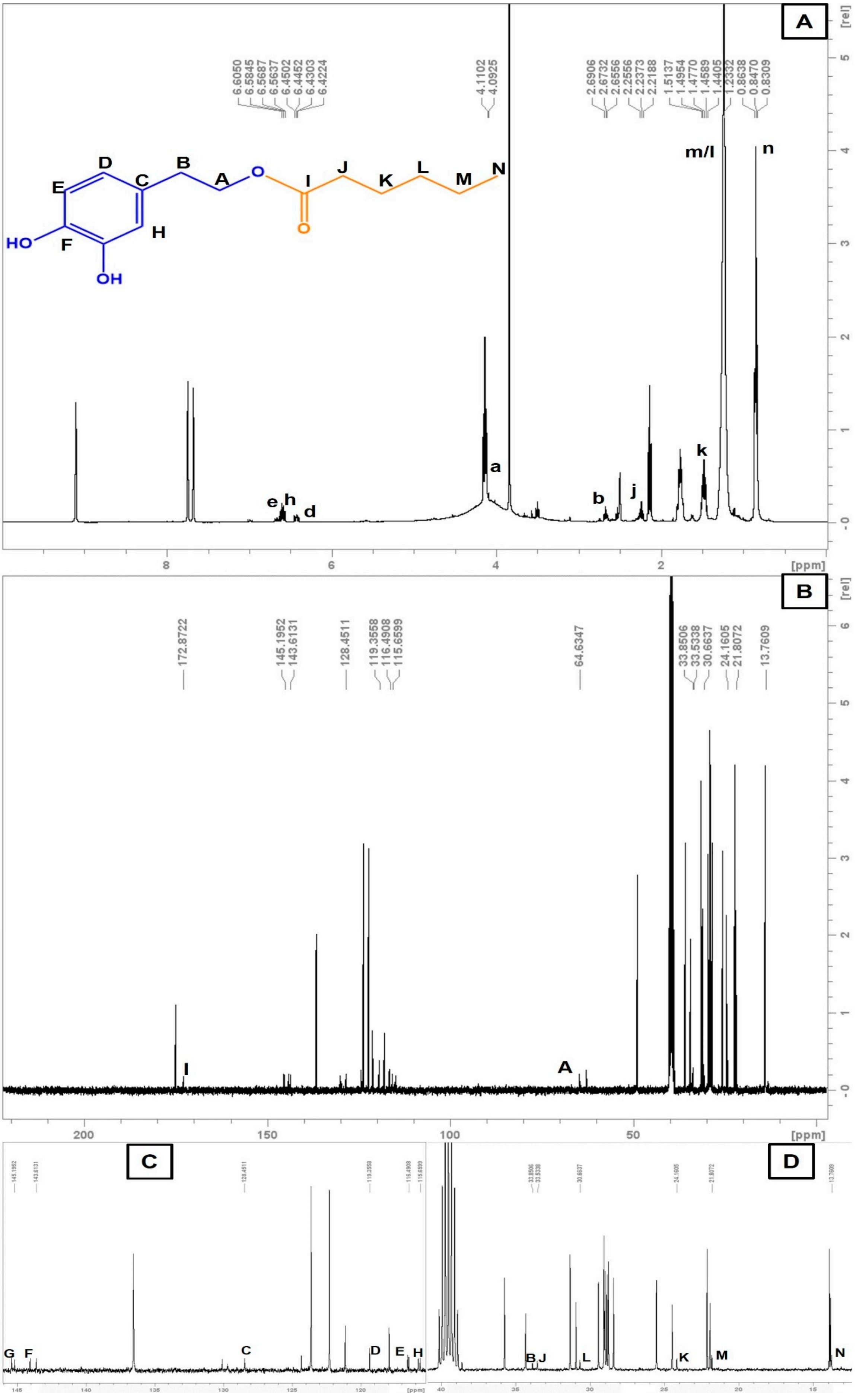

A
[rel]
6.6050
6.5845
6.5687
6.5637
6.4502
6.4452
6.4303
6.4224
4.1102
4.0925
2.6906
2.6732
2.6556
2.2556
2.2373
2.2188
1.5137
1.4954
1.4770
1.4589
1.4405
1.2332
0.8638
0.8470
0.8309
D
B
C
A
O
I
J
K
L
M
N
E
H
HO
F
OH
O
m/l
n
a
k
b
j
e
h
d
[ppm]
B
[rel]
172.8722
145.1952
143.6131
128.4511
119.3558
116.4908
115.6599
64.6347
33.8506
33.5338
30.6637
24.1605
21.8072
13.7609
I
A
[ppm]
C
G
F
C
D
E
H
[ppm]
D
B
J
L
K
M
N

Figure S7. ^{1}H-NMR (A) and ^{13}C-NMR (B) spectra of the reaction medium of HT-Hex synthesis. (C) and (D) are expanded regions of ^{13}C-NMR (B) spectra. Reaction performed with 1:4 HT:Hex (mol:mol), 80 ºC, 1h, in the SLIL [C_{12}mim][NTf_2] 70 % (w/w).

Hydroxytyrosyl hexanoate: ^{1}H-NMR δ(ppm):4.11 (t, 2H, H_a); 2.67 (t, 2H, H_b); 6.43 (dd, 1H, H_d); 6.59, (d, 1H, H_e); 6.56 (d, 1H, H_h); 2.23 (t, 2H, H_j); 1.47 (q, 2H, H_k); 1.18-1.33 (m, 4H, H_l and H_m, indistinguishable); 0.85 (t, 3H, H_n). ^{13}C-NMR δ(ppm): 64.6 (C_A); 33.8 (C_B); 128.5 (C_C); 119.4 (C_D); 116.5 (C_E); 143.6 (C_F); 145.1 (C_G); 115.7 (C_H); 172.9 (C_I); 33.5 (C_J); 24.1 (C_K); 30.7 (C_L); 21.8 (C_M); 13.8 (C_N).

2. Determination of residual IL in extracted HT-Hex by ^{19}F-RMN.

The esterification of HT at high scale was selected to perform the extraction of the HT-Hex product. A liquid-liquid extraction with five volumes of a mixture of PG:H_2O (85:15 v/v) was carried out, followed by cooling at 0 ºC and centrifuging to precipitate the solid IL. A 40 µL aliquot of the upper liquid phase containing the reaction species was analyzed by ^{19}F-NMR using 40 µL TFA as internal standard. Data were referred to a sample of pure IL to determine the of residual IL. Data reveals a residual IL content of only 1 %.

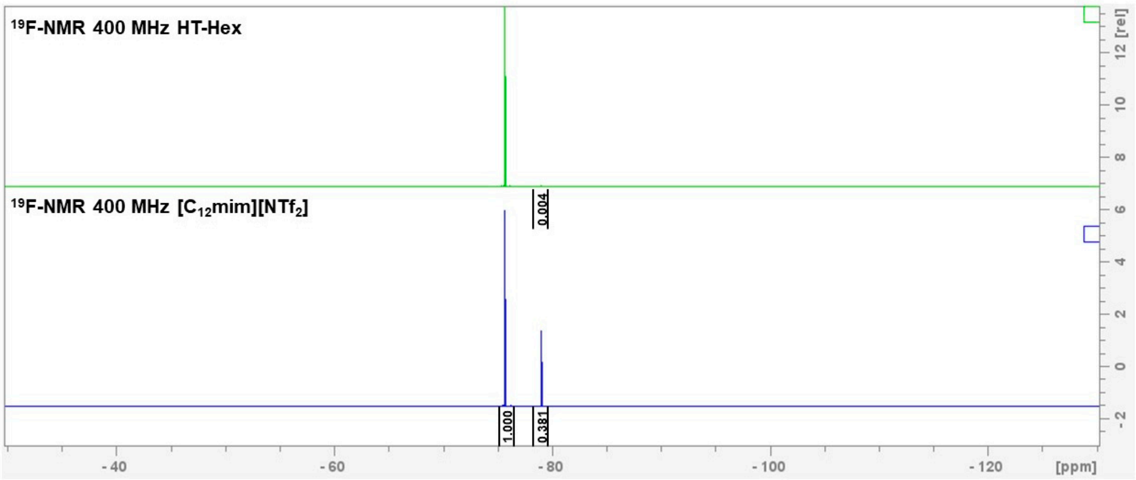

Figure S8. ^{19}F-NMR spectra of [C_{12}mim][NTf_2] and the extracted sample of HT-Hex.

3. Sustainable analysis of the synthesis of HT esters

Different green metric parameters have been selected to perform the analysis of sustainability. Table S1 collects those parameters, their formulas ad a brief definition.

Table S1. Green Metric Parameters, equations, and definitions. [1,2]

Parameter	Calculation	Definition
**Atom Economy (AE)**	$\mathrm{AE} = \frac{Products\ Mw}{\Sigma\ \mathrm{Reactants\ Mw}}$	Quantifies the atoms incorporated into the final product and the amount of waste produced in a reaction
**Stoichiometric Factor (SF)**	$\mathrm{SF} = 1 + \frac{Mass\ of\ Excess\ of\ Reactives\ (g)}{Mass\ of\ Stoichiometric\ Reactives\ (g)}$	Refers to the molar ratio of substrates and permits to perform calculations when using one or more reactants in excess with respect to a limiting one
**Yield of Panthenol Monoesters (ε)**	$\varepsilon = \frac{\mathrm{MSP}}{\mathrm{MMP}}$	Provides an insight into the reactivity of substrates and quantifies the selective (bio)catalytic productivity of the reaction
**Material Recovery Parameter[b] (MRP)**	$\mathrm{MRP} = \frac{1}{1 + \frac{\varepsilon \cdot\ \mathrm{AE} \cdot\ (\mathrm{C} + \mathrm{S} + \mathrm{W})}{\mathrm{SF} \cdot \mathrm{MSP}}}$	Shows the loss of auxiliary materials not recovered along the reaction and downstream steps
**Reaction Mass Efficiency (RME)**	$RME = \frac{\varepsilon\ \cdot \mathrm{AE}\ \cdot \mathrm{MRP}}{\mathrm{SF}}$	Mass-based metric, similar to AE, but also considers yield and use of excess reagents
**Product Mass Intensity (PMI)**	$\mathrm{PMI} = \frac{1}{\mathrm{RME}}$	Analyzes the input-output mass balance to identify the main sources of waste
**E-factor**	$E = \frac{1 - \mathrm{RME}}{\mathrm{RME}} = PMI - 1$	Quantifies the amount of waste formed per kg product
**Total Carbon Release (TCR)**	$\mathrm{TCR} = (\mathrm{PMI\ organic\ x\ 2.3}) + (\mathrm{PMI\ water\ x\ 0.63})$	Measures CO_2 emissions in case of waste incineration from organic and aqueous sources

Mw: molecular weight; MSP: Mass of synthesized products; MMP: Maximum mass of products; C: Catalyst (g); S: Substrates (g); W: Wastes (g) The term W does not include biocatalysts and LI because both are recovered.

Table S2. List of penalties assigned in each category of the EcoScale. The penalties are subtracted from an initial value of 100. F: Flammable; N: Dangerous for environment; T: Toxic; N.A.: Data not available.

**Entry**	**1**	**2**	**3**	**4**
Yield	-1	-6	-12.5	-12
Price availability				
Hydroxytyrosol	-5	-5	-5	-5
Ethyl palmitate	-5			
Vinyl decanoate		-5		
Hexanoic acid			0	0
MBTE		-5	-5	
CH_2Cl_2		N.A.		
n-Hexane	-5	N.A.	-3	
Acetonitrile	-5			
Propylene glycol				0
Safety*				
n-Hexane (F,N)	-10	-10	-10	
Acetonitrile (F,T)				
Ethanol (T,F)			-10	
Technical setup				
Common setup	0	0	0	0
Temperature-Time				
Heating > 1h	-3	-3	-3	-3
Work up and purification				
Classical chromatography	-10	-10		
Simple filtration	0	0		
Liquid-liquid extraction			-3	-3
Removal of solvent with bp < 150 °C	0	0		0
**EcoScale value**	**46**	**56**	**48.5**	**77**

1. Nieto, S., Bernal, J.M., Villa, R., Garcia-Verdugo, E., Donaire, A., Lozano, P. Sustainable set-ups for the biocatalytic production and scale-up of panthenyl monoacyl esters under solvent-free conditions. ACS Sustain. Chem. Eng. 2023, 11, 5737–5747, DOI: 10.1021/acssuschemeng.3c00266.

2. Sheldon, R. A. Metrics of Green Chemistry and Sustainability: Past, Present, and Future. ACS Sustainable Chem. Eng. **2018**, 6, 32−48, DOI: 10.1021/acssuschemeng.7b03505.
